# Altered hippocampal volume and functional connectivity in males with Internet gaming disorder comparing to those with alcohol use disorder

**DOI:** 10.1038/s41598-017-06057-7

**Published:** 2017-07-18

**Authors:** Eun Jin Yoon, Jung-Seok Choi, Heejung Kim, Bo Kyung Sohn, Hee Yeon Jung, Jun-Young Lee, Dai-Jin Kim, Sun-Won Park, Yu Kyeong Kim

**Affiliations:** 1 Department of Nuclear Medicine, Seoul Metropolitan Government Seoul National University Boramae Medical Center, Seoul, 07061 Republic of Korea; 2 Institute of Radiation Medicine, Medical Research Center, Seoul National University, Seoul, 03080 Republic of Korea; 3 Department of Psychiatry, Seoul Metropolitan Government Seoul National University Boramae Medical Center, Seoul, 07061 Republic of Korea; 4 Department of Psychiatry and Behavioral Science, Seoul National University College of Medicine, Seoul, 03080 Republic of Korea; 5 Department of Psychiatry, Seoul St. Mary’s Hospital, The Catholic University of Korea College of Medicine, Seoul, 06591 Republic of Korea; 6 Department of Radiology, Seoul Metropolitan Government Seoul National University Boramae Medical Center, Seoul, 07061 Republic of Korea; 7 Department of Radiology, Seoul National University College of Medicine, Seoul, 03080 Republic of Korea; 8 Department of Nuclear Medicine, Seoul National University College of Medicine, Seoul, 03080 Republic of Korea

## Abstract

Internet gaming disorder (IGD) has been conceptualized as a behavioral addiction and shares clinical, neuropsychological, and personality characteristics with alcohol use disorder (AUD), but IGD dose not entail brain exposure to toxic agents, which renders it different from AUD. To achieve a clear understanding of the neurobiological features of IGD, we aimed to identify morphological and functional changes in IGD and compare them with those in AUD. Individuals with IGD showed larger volume in the hippocampus/amygdala and precuneus than healthy controls (HCs). The volume in the hippocampus positively correlated with the symptom severity of IGD. Moreover, functional connectivity analysis with the hippocampus/amygdala cluster revealed that the left ventromedial prefrontal cortex showed stronger functional connectivity in individuals with IGD compared to those with AUD. In contrast, individuals with AUD exhibited the smaller cerebellar volume and thinner medial frontal cortex than HCs. The volume in the cerebellum correlated with impaired working memory function as well as duration of illness in AUD group. Findings suggested that altered volume and functional connectivity in the hippocampus/amygdala in IGD might be associated with abnormally enhanced memory process of gaming-related cues, while abnormal cortical changes and cognitive impairments in AUD might be associated with neurotoxic effects of alcohol.

## Introduction

Internet gaming disorder (IGD) is defined as the excessive or uncontrolled Internet gaming activity, leading to negative consequences in the psychosocial function^1^. As IGD has been considered to be a significant public health issue, the Diagnostic and Statistical Manual of Mental Disorders, fifth-edition (DSM-V) recently included IGD as a condition for further study^2^. IGD has been conceptualized as a behavioral addiction and shares neuropsychological characteristics with substance use disorders (SUD) including cravings, withdrawal during abstinence, and tolerance^3^. In addition, impaired decision-making and inhibitory control that are key features of SUD are frequently observed in individuals with IGD^4, 5^. Therefore, the underlying neural mechanisms of IGD are hypothesized to overlap with those of SUD. However, IGD dose not entail brain exposure to toxic agents, which renders it different from SUD. To achieve a clear understanding of the neurobiological features of IGD and to establish IGD as a formal disorder, we need to directly compare the brain changes in IGD with those in SUD.

Recently, several cortical morphometry studies have found possible neural mechanisms underlying IGD. The brain regions associated with impulsivity and executive control, such as the orbitofrontal cortex, dorsolateral prefrontal cortex (DLPFC), and anterior cingulate cortex have consistently shown decreased gray matter volume^6–9^ and thinner cortical thickness^10, 11^ in IGD individuals comparing to healthy controls (HCs). Moreover, the changes in these brain regions showed correlations with disease severity^6, 8^ or cognitive control deficit^9, 11^, which support the important role of the prefrontal area in IGD. In addition to prefrontal area, previous studies have also shown changes in other brain system, such as reward, learning and memory and motor and sensory system. IGD individuals showed decreased volume in supplementary motor area and visual association area^6, 7, 9^, increased volume in the thalamus^12^, and increased cortical thickness in the precentral and middle temporal cortices^11^, which might be associated with excessive motor and sensory processing during Internet game play. Several studies have also found decreased gray matter volume in the amygdala^13^ and striatum^14^ as a part of brain reward system, and increased cortical thickness in the precunues that is associated attention and memory retrieval^11^. Gaming experience also associated with structural changes in healthy subjects. The amount of lifetime video gaming was positively associated with bilateral hippocampal, parahippocampal and occipital volume^15^. In addition, gray matter volume in the right hippocampus and DLPFC significantly increased after intense video gaming training and these increased volume correlated with desire to paly game^16^. These results suggested that the long-time gaming exposure might be related with changes in brain memory and executive control system, and these changes could be a possible mechanism of excessive game playing.

Findings in recent several functional neuroimaging studies comparing between IGD and alcohol use disorder (AUD) are in line with the previous brain structure studies of IGD. AUD, a representative type of SUD, shares features related to emotion, temperament, and personality^17^ as well as aforementioned behavior symptoms with IGD. Both IGD and AUD groups showed increased regional homogeneity in the posterior cingulate cortex^18^, and positive functional connectivity in the cingulate gyrus and cerebellum, and negative functional connectivity in the orbitofrontal cortex on the DLPFC-seeded functional connectivity analysis^19^. These results suggest that both IGD and AUD groups may have common problems with control of response inhibition and executive function. On the other hand, IGD also showed reduced regional homogeneity in the superior temporal gyrus^18^, and lower absolute beta power than AUD group and HCs^20^. On the DLPFC-seeded functional connectivity analysis, individuals with IGD showed positive functional connectivity in the striatum and temporal area, while individuals with AUD showed negative functional connectivity in these areas^19^. These differences were suggested as results of excessive processing of audio and visual gaming information in IGD. Taken together, not only did these functional neuroimaging studies comparing IGD to AUD demonstrated that IGD shared neurobiological mechanisms with AUD, but they also clarified unique characteristics of functional brain changes associated with gaming experience in IGD. However, there is no brain morphological study directly comparing between IGD and AUD to support the potential neurobiological marker in IGD identified in the previous functional imaging studies.

Therefore, the aim of this study was to investigate the neurobiological mechanisms of IGD by examining the structural cortical changes using various views, comparing them with those of AUD and HCs, and observing the relationship between the cortical changes and neurocognitive function. Additionally, we evaluated the functional connectivity of the brain regions showing significant morphological changes in IGD. It is unclear whether gray matter volume alterations are directly related to altered functional connectivity within brain networks. However, recent studies with psychiatric disorders identified alterations of resting-state functional connectivity using altered gray matter volume as the seed ROI, which provided more comprehensive understanding of the underlying neural circuitry of disorders than when only morphological changes investigated^21, 22^. We hypothesized that both IGD and AUD individuals would show cortical abnormalities associated with executive control, such as the prefrontal areas and IGD would also show cortical abnormalities associated with processing and learning of gaming-related information, such as the motor and visual area, and the hippocampus.

## Results

### Participant characteristics

The demographic and clinical characteristics of the participants are presented in Table 1. All participants were older than18 years of age, but the IGD group was significantly younger than the AUD group. The subjects with AUD obtained lower intelligence quotient (IQ) scores than the HCs. Both the IGD and AUD groups obtained higher scores than HCs on the Beck Depression Inventory (BDI), the Beck Anxiety Inventory (BAI), and the Barratt Impulsiveness Scale-11 (BIS-11). No significant differences were found between the IGD and the AUD groups with respect to BDI, BAI, and BIS-11 scores and duration of illness. There were no significant differences in smoking habit between 3 groups. None of the participants with addiction qualified other psychiatric disorders according to the Structured Clinical Interview for DSM-IV (SCID).

**Table 1:** Demographic and clinical characteristics and neuropsychological performance of participants.

	Internet gaming disorder (IGD)	Alcohol use disorder (AUD)	Healthy control (HC)	Group comparisons		
				Test statistics^†^	*p*	Post Hoc
Number (n)	19	20	25			
Age (years)^a^	22.9 ± 5.2	28.7 ± 5.8	25.4 ± 3.8	6.70	0.002	IGD < AUD^***^
IQ^a^	116.6 ± 13.4	112.0 ± 11.6	121.4 ± 9.2	3.88	0.026	AUD < HC^*^
IAT	76.3 ± 7.8	29.6 ± 5.7	26.0 ± 13.1	39.86	<0.001	IGD > AUD^****^, HC^****^
AUDIT-K	4.0 ± 2.8	24.0 ± 5.3	5.9 ± 3.3	42.34	<0.001	AUD > IGD^****^, HC^****^
BDI	19.4 ± 9.3	25.6 ± 14.5	3.6 ± 3.3	40.08	<0.001	HC < IGD^****^, AUD^****^
BAI	17.7 ± 14.7	23.9 ± 15.4	6.2 ± 5.6	18.82	<0.001	HC < IGD^***^, AUD^****^
BIS-11	70.1 ± 11.8	75.4 ± 14.6	54.3 ± 9.2	25.74	<0.001	HC < IGD^****^, AUD^****^
Smoking (smoker/non-smoker)^b^	3/16	5/15	5/20		0.861	
Duration of illness (years)^c^	6.3 ± 2.8	5.4 ± 2.7		152.50	0.134	
Neuropsychological tests						
VF total correct response^a^	43.5 ± 13.8	37.3 ± 12.2	45.5 ± 12.3	2.40	0.10	—
Stroop color and word test						
Subtraction score^a,d^	40.0 ± 14.2	40.8 ± 20.2	35.48 ± 16.1	0.65	0.52	—
Color reading errors	1.8 ± 1.8	1.5 ± 1.7	2.4 ± 2.0	3.47	0.18	—
TMT-A (sec)	22.5 ± 9.0	21.8 ± 6.5	18.8 ± 6.3	3.49	0.17	—
TMT-B (sec)	54.4 ± 22.2	54.4 ± 14.8	53.56 ± 30.9	1.40	0.51	—
IED total errors	12.6 ± 11.0	19.3 ± 17.6	16.2 ± 16.9	4.61	0.10	—
SOC problems solved in minimum moves	9.2 ± 2.2	7.9 ± 2.0	8.2 ± 1.8	4.02	0.13	—
Spatial span						
Span length	8.1 ± 1.4	7.2 ± 1.4	8.0 ± 1.8	8.34	0.02	AUD < HC^**^
Total errors	6.5 ± 6.6	13.0 ± 5.3	9.5 ± 8.0	9.29	0.01	IGD < AUD^***^

IQ, intelligence quotient; IAT, Young’s Internet Addiction Test; AUDIT-K, Korean version of the Alcohol Use Disorder Identification Test; BDI, Beck Depression Inventory; BAI, Beck Anxiety Inventory; BIS-11, Barratt Impulsiveness scale-11; VF, verbal fluency; TMT, Trail Making Test; IED, Intra-Extra Dimensional Set Shift; SOC, Stockings of Cambridge. ^†^Kruskal-Wallis test was used for group comparisons with Mann-Whitney U post-hoc tests and *χ* ^2^ is reported unless a footnote appears at the name of the parameter. ^a^One-way ANOVA was used for group comparison with Tukey’s HSD post-hoc tests and F ratio is reported. ^b^Fisher’s exact test was used for group comparison. ^c^Mann-Whitney U test was used for group comparison and value of U is reported. ^d^The time for the color-word condition minus that for the word condition. *p < 0.05 **p < 0.01 ***p < 0.005 ****p < 0.001.

### Differences in neuropsychological function

The results of the neuropsychological tests are presented in Table 1. We found significant differences in span length and total errors on the Spatial Span test (SSP). The post-hoc test revealed that the individuals with AUD exhibited shorter span length than HCs and committed more total errors than the individuals with IGD. There were no significant differences between groups in any of the other neuropsychological tests.

### Differences in gray matter volume

**Figure 1:**
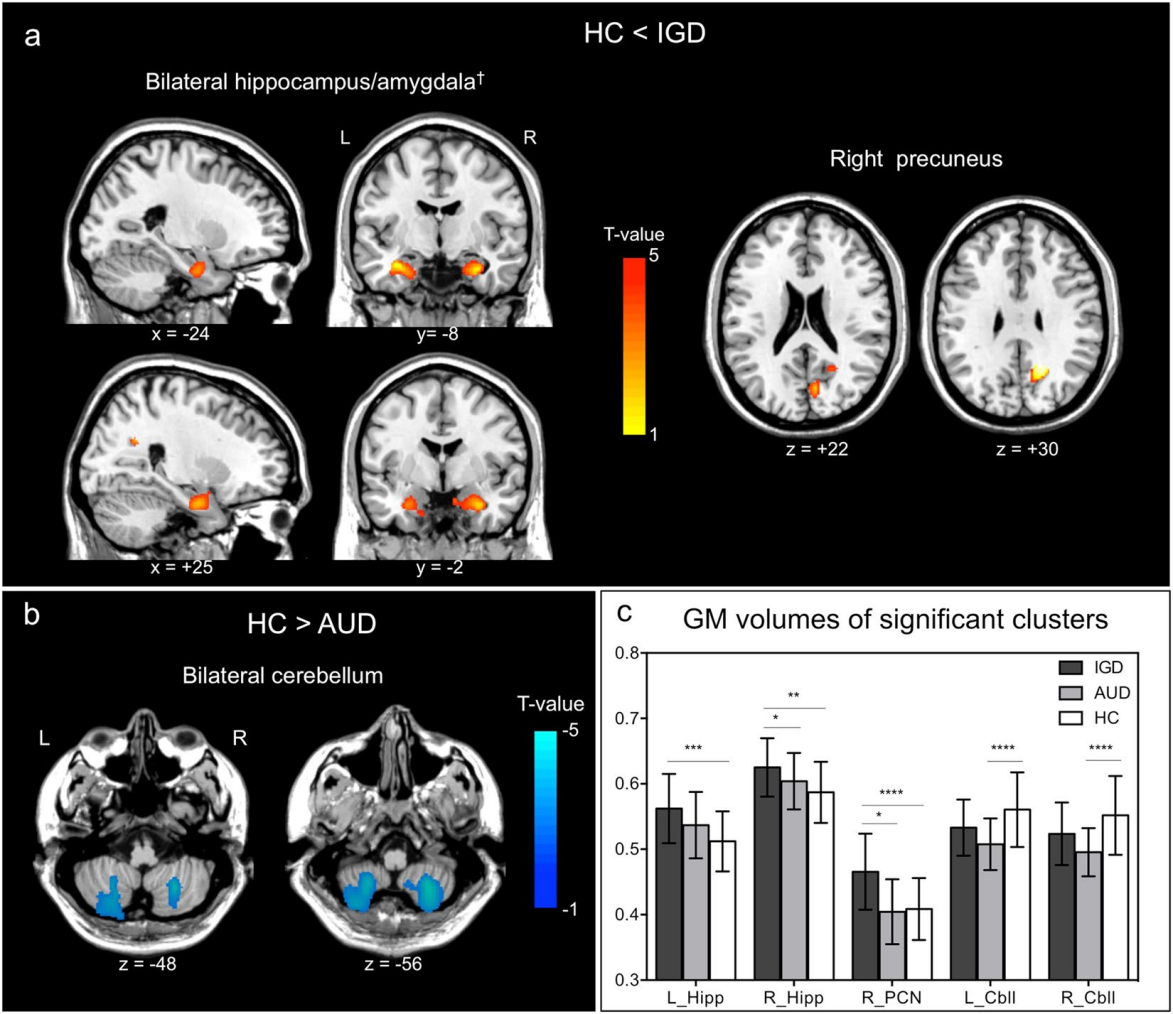
Results of cortical volume comparisons among Internet gaming disorder (IGD), alcohol use disorder (AUD), and healthy controls (HCs). (**a**) Areas of significant larger gray matter volume in IGD than HCs. ^†^The cluster size of the right hippocampus/amygdala did not reach the level of significace (883 voxels; minimum cluster size for significance = 953 voxels). (**b**) Areas of significant smaller gray matter volume in AUD than HCs. (**c**) Average gray matter volumes of the significant clusters for each group. Error bars denote standard error of the mean. *p < 0.05; **p < 0.01; ***p < 0.005; ****p ≤ 0.001; HC, healthy control; AUD, alcohol use disorder; IGD, Internet gaming disorder; L, left; R, right; L_Hipp, left hippocampus; R_Hipp, right hippocampus; R_PCN, right precuneus; L_Cbll, left cerebellum; R_Cbll, right cerebellum.

The IGD group exhibited lager volume in the left hippocampus/amygdala and right precuneus than HCs (minimum cluster size for significance = 953 voxels). The right hippocampus/amygdala in the IGD group also showed lager volume, although the cluster size did not reach the level of significance (883 voxels) (Fig. 1a, Table 2). In contrast, the AUD group showed reduced volume in the bilateral cerebellum compared with controls (minimum cluster size = 973 voxels) (Fig. 1b, Table 2). Comparisons between the IGD and the AUD groups revealed no significant differences in cortical volume.

**Table 2:** Brain regions showing significant changes in cortical volume among Internet gaming disorder, alcohol use disorder and healthy controls groups.

Region	Peak MNI coordinates			t-value	z-value	Size (voxels)	p-values at peak level	p-values at cluster level
	x	y	z					
**Internet gaming disorder > Healthy controls**								
Rt precuneus (BA 31)	16	−56	34	5.46	4.88	959	<0.001	0.015
Lt hippocampus/amygdala	−34	−9	−21	4.17	3.89	989	<0.001	0.014
Rt hippocampus/amygdala^a^	30	−4	−26	4.11	3.84	883	<0.001	0.019
**Alcohol use disorder** < **Healthy controls**								
Lt Cerebellum (tonsil and inferior semi-lobar lobule)	26	−60	−51	3.93	3.69	2558	<0.001	<0.001
Rt Cerebellum (tonsil and inferior semi-lobar lobule)	−24	−62	−52	3.75	3.53	3100	<0.001	<0.001

Lt, left; Rt, right; BA, Brodmann area. ^a^The cluster size did not reach significant level.

### Differences in cortical thickness

**Figure 2:**
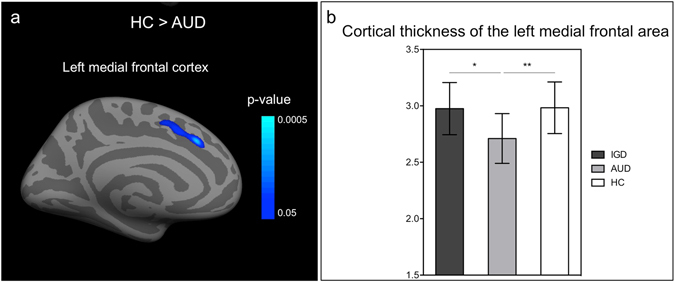
Result of cortical thickness analysis. (**a**) Significance maps of the group differences in cortical thickness in the participants with alcohol use disorder compared to healthy controls. (**b**) Average cortical thicknesses of the significant cluster for each group. Error bars denote standard error of the mean. *p ≤ 0.005; **p < 0.001 HC, healthy control; AUD, alcohol use disorder; IGD, Internet gaming disorder.

Individuals with IGD did not show significant differences in cortical thickness compared with IGD group or HCs. The AUD group exhibited thinner left medial frontal cortex and the adjacent anterior midcingulate cortex (aMCC) relative to the HCs (peak MNI coordinates, x = −10, y = 24, z = 37; cluster-wise *p* = 0.04) (Fig. 2).

### Relationship between brain cortical changes and clinical characteristics

**Figure 3:**
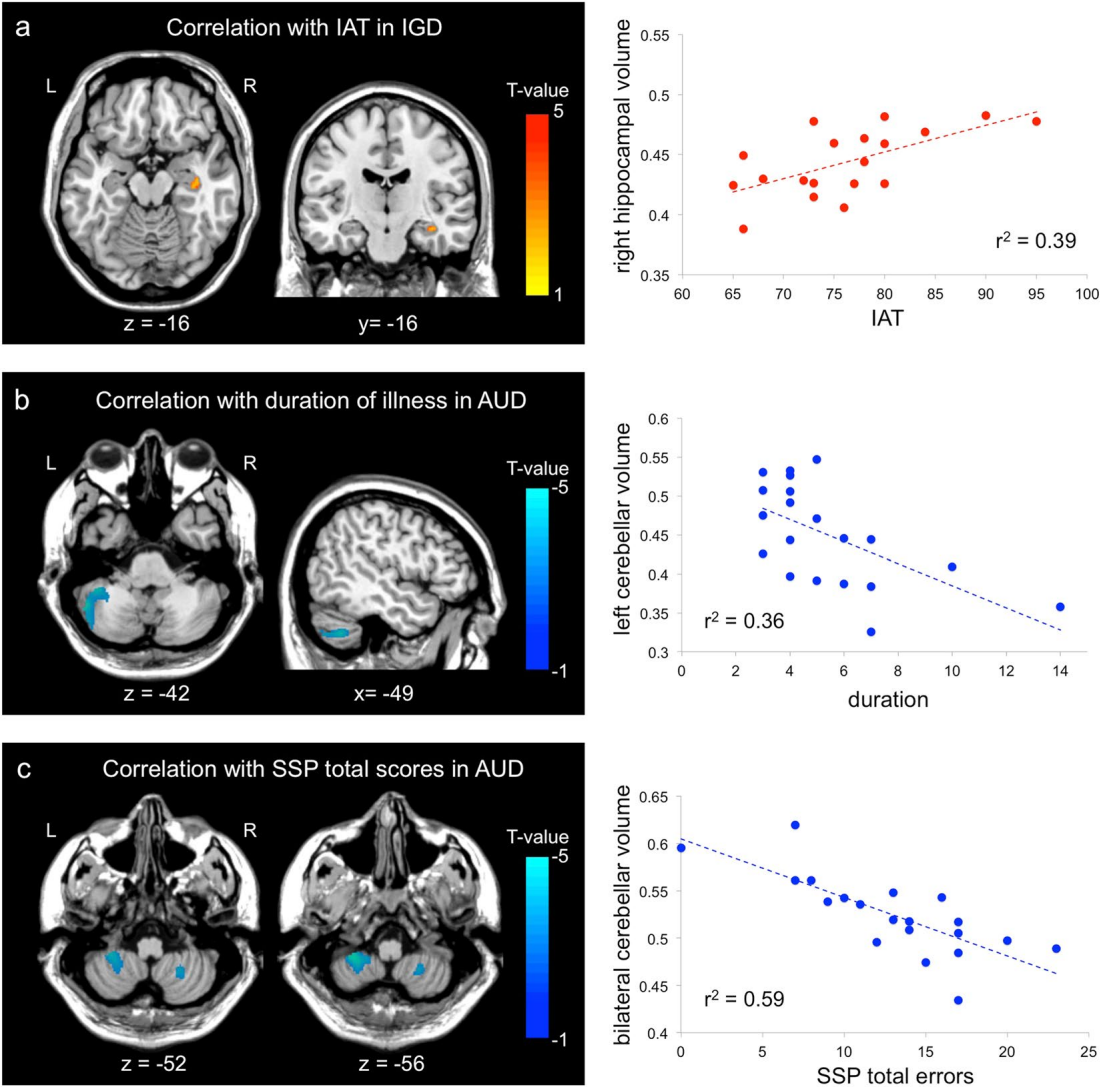
Brain regions showing significant correlations between gray matter volume and clinical characteristics or cognitive function. (**a**) The brain region showed positive correlations with scores of Young’s Internet addiction test in Internet gaming disorder group (IGD). The scatter plot at right depicts the relation between mean gray matter volume from the significant cluster and IAT scores in IGD. (**b**) The brain regions showed negative correlation with addiction duration of alcohol use disorder (AUD). The scatter plot at right depicts the relation between mean gray matter volume from the significant cluster and addiction duration of AUD. (**c**) The brain regions showed negative correlation with total errors of spatial span in AUD group. The scatter plot at right depicts the relation. These correlations were not significant after correctios for multiple comparisons. between mean gray matter volume from the significant cluster and total errors of spatial span in AUD group. AUD, alcohol use disorder; IGD, Internet gaming disorder; IAT, Young’s Internet addiction test; SSP, spatial span.

The gray matter volume in the right hippocampus of the individuals with IGD was positively correlated with their Young’s Internet Addiction Test (IAT) scores (*r* = 0.63, *p* = 0.004) (Fig. 3a). For the AUD group, left cerebellar volume was negatively correlated with illness duration (*r* = −0.60, *p* = 0.006) (Fig. 3b), and bilateral cerebellar volume was negatively correlated with total errors on the SSP (*r* = −0.77, *p* < 0.001) (Fig. 3c). These correlations were not significant after corrections for multiple comparisons. Any clinical characteristics did not show correlations with cortical thickness changes.

### Functional connectivity pattern of the hippocampus/amygdala

**Figure 4:**
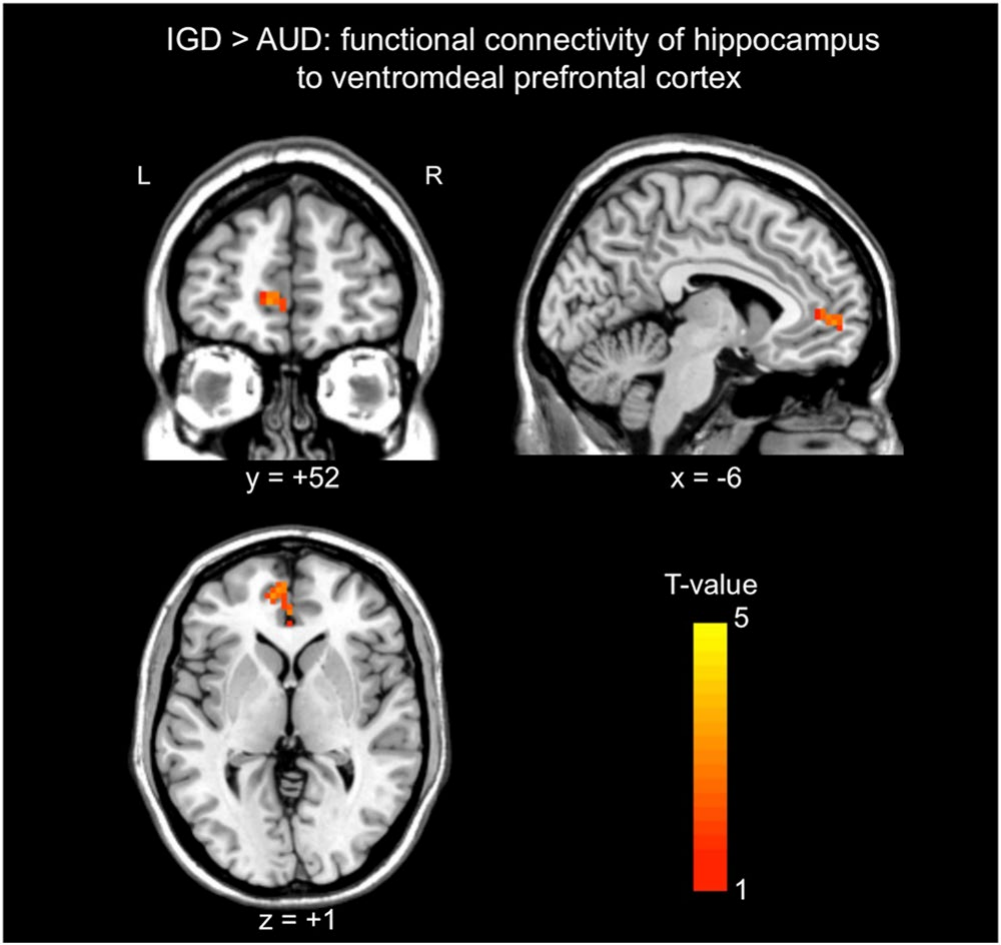
Results of functional connectivity anaysis with a seed region consisting of the hippocampus/amygdala showing volume differences in individuals with Internet gaming disorder (IGD) compared to those with alcohol use disorder (AUD). The left ventromedial prefrontal cortex showed stronger fuctional connectivity in the individualss with IGD compared to those with AUD. AUD, alcohol use disorder; IGD, Internet gaming disorder.

The bilateral hippocampus/amygdala clusters of the VBM analysis was defined as seed ROI for the resting-state functional connectivity analysis, because this region also showed significant correlation with IAT, and their essential roles in the memory and learning of addiction-related cues is suggested^23, 24^. There were no brain regions showing significant differences in functional connectivity with the hippocampus/amygdala between HCs and each addiction group, AUD and IGD. However, the individuals with IGD showed significant stronger functional connectivity with the hippocampus/amygdala region to the left ventromedial prefrontal cortex (vmPFC) than the individuals with AUD (peak MNI coordinates, x = −9, y = 48, z = 3; cluster size = 44 voxles; minimum cluster size for significance = 42 voxels) (Fig. 4).

## Discussion

In this study, we identified individuals with IGD had lager volume in the bilateral hippocampus/amygdala and right precuneus than HCs. Also, the volume of the right hippocampus was positively correlated with IAT scores. Considerable evidences suggest that the hippocampus and amygdala play a critical role in the acquisition, consolidation, and retrieval of learning of addiction-related cues that leads to compulsive addictive behavior^23, 24^. Lesioning or pharmacological inactivation of either the hippocampus or amygdala diminished cue-induced drug-seeking behaviors^25^, and disconnection between the amygdala and hippocampus inhibited reconsolidation of cocaine-related associative memories^26, 27^. Human neuroimaging studies have found that cue-induced cravings are associated with increased activation in the amygdala and hippocampus among cocaine-dependent subjects^28^ and pathological gamblers^29^. Consistent findings have been also reported in the IGD. Individuals with IGD have exhibited increased activity in the hippocampus and parahippocampal gyrus triggered by gaming cues, and this activity was correlated with the self-reported desire for games^30–34^. More recent studies have found structural changes in the hippocampus related to gaming experience. The amount of lifetime video gaming was positively correlated with the bilateral hippomcapal and parahippocampal volume^15^, and gray matter volume in the right hippocampus significantly increased after video gaming training in HCs^16^. Jeong *et al*.^35^ found increased fractional anisotropy (FA) in the broad white matter area including the right cingulum to the hippocampus, and this increased FA correlated with duration of illness among IGD subjects.

Moreover, we identified significantly stronger functional connectivity between the hippocampus/amygdala and the left vmPFC in individuals with IGD than those with AUD. The vmPFC is core regulatory region of emotion and reward processing^36, 37^. In individuals with online gaming addiction, the vmPFC showed increased gaming-cue related activity, and the activation was positively correlated with gaming urge^38^. It is known that the vmPFC and hippocampus are coupled with each other to mediate memory-guided choices and retrieval-mediated learning process^39, 40^. Internet gaming provides diverse content and information using multisensory stimuli in novel situations and creates high arousal and sensory overload. Moreover, game playing requires an active working memory system and sustained visual and auditory attention. Therefore, the lager volumes in the hippocampus/amygdala, its significant correlation with symptom severity, and stronger functional connectivity with the vmPFC that were observed in the present study might be associated with experience-dependent changes related to stronger memories for Internet game-related cues or responses subsequent to Internet gaming, or they may reflect factors that render individuals vulnerable to frequent or strong cravings for Internet gaming.

Similar with the hippocampus, the precuneus contributes to cue-induced cravings in IGD^32–34^ as well as SUD^28^ and pathologic gambling^29^. Previous studies regarding online gaming addiction have found the precuneus hyperactivity in response to online gaming cues; this activity increase was correlated with gaming-related cravings, urges, and the severity of the online gaming addiction^32, 33^. Yuan *et al*.^11^ identified that individuals with IGD exhibited an increased cortical thickness in the precuneus, with this increase reflecting a correlation with the duration of IGD. It is known that the precuneus participates in visual–spatial information processes and episodic memory retrieval^41^. Additionally, as the functional core of the default mode network, the precuneus is implicated in attention to external environment^42, 43^. Therefore, the larger volume of the precuneus in IGD might be associated with abnormal attentional orientation to the Internet gaming-related cues and memories.

It has been suggested that impairments of prefrontal functions, in particular executive control over behavior, contribute to a loss of control over Internet use^44^. Previous neuroimaging studies have repeatedly reported structural changes in the prefrontal cortex, supporting the contributing role of the prefrontal cortex to IGD^6–8, 10, 11^. In the present study, however, there were no significant reduced volume or thickness in the prefrontal cortex in the individuals with IGD. Moreover, our IGD individuals showed normal performance on executive function tests. Previous researches with IGD showing significantly decreased volume or thickness in the prefrontal cortex were studied in adolescents^6, 10^ or average of 19 years old with standard deviation of 1–2 years^7, 9, 11^. While, the mean age of IGD individuals in the present study was 22.9 ± 5.2 years old. Other VBM studies with IGD subjects of the similar ages with ours did not find significant changes in the prefrontal area^12, 13^. Adolescence is a critical neurodevelopmental period, with maturation of gray- and white matter and age-related increases in cognitive function and social behavior^45–47^. Therefore, it is possible that adolescents have different neural substrate of IGD from young adults. Particularly, the prefrontal cortex is the last cortical region to reach full gray matter maturation in early adulthood^48^, so Internet gaming might affect more immature prefrontal cortex in adolescence than prefrontal cortex in adults. The different brain morphological changes between adolescence and adults with IGD should be addressed in the further studies. Meanwhile, there were only two cortical thickness studies with IGD, and the results have some contradiction each other^10, 11^, therefore, it is suggested that the cortical thickness is not robust measurement for evaluation of brain changes in IGD^49^. In line with this suggestion, present study did not found significant differences in IGD compared to HD or AUD. However, these three studies differ in the age of participants and have relative small number of participant (>20). Therefore, further studies both in adolescence and adult with large number of IGD are needed to confirm the cortical thickness changes in IGD.

On the other hand, the individuals with AUD as compared with HCs had significantly smaller volume in the bilateral cerebellum and thinner left medial frontal cortex along with the adjacent aMCC, which is consistent with previous studies focusing on brain structural changes in AUD. Early postmortem brain and neuroimaging studies have consistently shown alcohol-related reductions in the number of neurons and in the cortical volume, with marked losses in the prefrontal and cerebellar areas^50–53^, and whole-brain morphometric studies of AUD also confirmed the brain abnormalities including the prefrontal and cerebellar regions^54–58^. Recent brain morphology studies have suggested that the neurotoxic effects of alcohol disrupt typical neurodevelopment in the prefrontal and cerebellar regions. Adolescents and young adults with adolescent-onset AUD had reduced volume in the prefrontal cortex compared with controls, and males with AUD additionally showed reduced volume in the cerebellar hemisphere^53^. Furthermore, reduced gray matter volume in the prefrontal area and cerebellum was correlated with the age at which adults with AUD started drinking^54^. Mashhoon *et al*.^59^ found reduced cortical thickness in the aMCC of emerging adults with binge-drinking relative to light-drinking adults, and the altered aMCC thickness was negatively correlated with the quantity and frequency of alcohol consumption. These results suggest that an early-onset of drinking impedes the maturation of the prefrontal and cerebellar regions. In the present study, the participants with AUD were younger than those in other studies of AUD, and all the AUD individuals had started to drink alcohol either during adolescence or young adulthood. Thus, the abnormalities in the cerebellum and medial frontal cortex that were found in the present study support the vulnerability of these regions to early-onset drinking. Additionally, we found reduced cerebellar volume correlated with duration of AUD. This finding might be consistent with the previous studies supporting neurotoxic effect of alcohol^51, 52^.

Regarding the alteration of the frontal regions in this study, however, no correlation between the medial frontal cortical thickness and duration of illness or disease severity was found. Recent a neuroimaging study identified that the aberrant fronto-cerebellar connectivity in alcohol-naïve youth with a family history of alcoholism, suggesting as a distinct vulnerability marker for AUD^60^. Therefore, it is also possible that the abnormal volume and thickness could be preexisting neurophenotypic factors of excessive alcohol intake rather than resulting from neurotoxic effects.

The cerebellar volume was negatively correlated with working memory performance in the AUD group. It is well documented that AUD is associated with cognitive impairments, such as reduced working memory, verbal memory, visuospatial abilities, and response inhibition^61^. In the present study, we identified impairment in the working memory function of individuals with AUD, as measured by the SSP, and found that the number of total errors on the SSP was correlated with reduced volume in the cerebellum. The correlation between working memory function and cerebellar volume is consistent with previous studies showing that the cerebellum is involved in executive functioning and that a disruption in frontocerebellar circuitry is the basis of the impaired cognitive performance of individuals with AUD^62^.

There are several limitations to the current study. The first limitation of this study is the between-group differences in basic demographic characteristics, such as age and IQ. However, we included age and IQ as nuisance variables in the brain cortical analyses, and the brain clusters that yielded between-group differences were not correlated with age or IQ. Second, because the present study recruited only male participants, there is a limitation to generalize the results to females. Studies with demographically well-matched groups including both male and female are needed to replicate and refine our findings. Finally, present study was cross-sectional study. Therefore, we did not infer the causal relationships between brain morphological and functional changes and addictive behaviors. Further longitudinal study is needed to confirm underlying mechanisms of brain changes in IGD and AUD.

In conclusion, larger volume in the hippocampus/amygdala and their stronger functional connectivity with prefrontal cortex in in individuals with IGD can characterize the pathophysiology of IGD, such as enhanced memory process of gaming-related cue, while abnormal cortical changes and cognitive impairments in individuals with AUD might be associated with neurotoxic effects of alcohol. The findings suggest that the brain morphological changes in IGD have their own characteristics that differ from those in AUD and that these changes may be implicated in the underlying pathophysiology of IGD.

## Methods

### Participants

We enrolled 19 individuals diagnosed with IGD, 20 individuals diagnosed with AUD, and 25 HCs between 2013 and 2015. Since males have a higher potential to be addicted to Internet gaming than females do^63, 64^, we recruited only males. All individuals with IGD or AUD were seeking treatment for excessive participation in Internet gaming or alcohol consumption and visited to the outpatient clinics of the SMG-SNU Boramae Medical Center. HCs were recruited through an advertisement posted on the SMG-SNU Boramae Medical Center.

IGD was diagnosed according to the DSM-IV criteria and the severity of IGD was assessed using IAT^65^. We included subjects with IAT scores of at least 50 who spent more than 4 hours per day and 30 hours per week involved in Internet gaming. The mean IAT score of the individuals with IGD was 76.3 ± 7.8, and their mean time spent on Internet gaming was 7.7 ± 3.5 hours per day.

AUD was diagnosed based on the SCID and, the severity of AUD was assessed using the Korean version of the Alcohol Use Disorder Identification Test (AUDIT-K)^66^. The mean AUDIT-K score of the individuals with AUD was 24.0 ± 5.3, and their mean quantity of alcohol consumed per day was 8.3 ± 2.5 standard drinks. Individuals with AUD were abstinent from alcohol at least 2 weeks before participation in the present study. Alcohol abstinence was confirmed self-reports and reports from caregivers. We regarded these reports as reliable because the participants attended regular follow-up visits to our outpatient clinic and showed good adherence to treatment.

The SCID was used to identify past and current psychiatric illnesses of addiction groups. Individuals with AUD and HCs used the Internet less than 2 hours per day and mean IAT score of AUD group was 29.6 ± 5.7, and that of HCs was 26.0 ± 13.1. Individuals with IGD and HCs had no lifetime history of AUD and drank fewer than 14 standard drinks per week and fewer than 4 standard drinks per occasion. Mean AUDIT-K of IGD was 4.0 ± 2.8 and, that of HCs was 5.9 ± 3.3.

Exclusion criteria included a history of significant head injury, seizure disorder, mental retardation, other substance abuse (except smoking) or psychotic disorder and IQ score estimated by the Korean version of the Wechsler Adult Intelligence Scale (WAIS-III)^67^ below 80. Individuals with both IGD and AUD were excluded from participation in this study. All participants completed the BDI^68^, BAI^69^, and BIS-11^70^. The study protocol was approved by the Institutional Review Board of the SMG-SNU Boramae Medical Center and all methods were performed in accordance with the approved guidelines and the Declaration of Helsinki. All subjects gave written informed consent before enrolling in this study after a detailed explanation of the procedure.

### Image acquisition

We used a 3.0 T MR system (Achieva, Philips Medical Systems, Best, The Netherlands) to acquire a high-resolution T1-anatomical brain image with a 3D T1-weighted turbo field echo (T1TFE) sequence (TR = 9.9 ms, TE = 4.6 ms, flip angle = 8°, field of view = 220 × 220, 180 slices, thickness = 1 mm, voxel size = 0.98 mm × 0.98 mm × 1 mm) and whole-brain resting-state functional image using a T2*-weighted echo-planar imaging sequence (TR = 2700 ms, TE = 35 ms, flip angle = 90°, field of view = 220 × 220, 35 slices, thickness = 4 mm, voxel size = 1.53 mm × 1.53 mm × 4 mm, 180 volumes). During functional MRI (fMRI) scanning, participants were instructed to rest with their eyes open, not to sleep, and not to think about anything in particular.

### Image processing

#### Cortical volume

VBM^71^ was performed using SPM8 (http//www.fil.ion.ucl.ac.uk/spm/software/spm8) implemented in Matlab 7.11 (The MathWorks, Inc. Natick, MA, USA). Each anatomical MR image was segmented and non-linearly normalized to a standard template using the DARTEL algorithm provided in SPM8. The spatially normalized images were then rescaled using the Jacobian determinants of the deformations to preserve relative tissue volumes and smoothed using an 8-mm full-width half-maximum (FWHM) Gaussian kernel.

#### Cortical thickness

FreeSurfer imaging analysis suite (http://surfer.nmr.mgh.harvard.edu/; version 5.1.0) was used to estimate cortical thickness. The local cortical thickness was measured on the basis of the difference between the position of equivalent vertices in the pial and gray–white matter surfaces. The details of these procedures have been extensively described in a prior publication^72^. Segmented volumes were visually inspected, and the appropriate manual corrections were performed. To obtain cortical thickness difference maps, the data were smoothed on the surface with a 10-mm FWHM Gaussian kernel.

#### Functional connectivity

Image preprocessing and statistical analysis were carried out using SPM8 and DPARSF (Data Processing Assistant for Resting-State fMRI, www.restfmri.net)^73^, based on MATLAB toolbox. The first 10 volumes of the functional time series were discarded. The remaining 170 volumes were subjected to slice timing correction, and to realigning with a six-parameter spatial transformation. Data from participants with head-motion exceeding 2.0 mm of maximal rotation and 2.0° of any angular motion during the scan were eliminated from the analysis. Data of 2 IGD subjects, 4 AUD subjects and 3 HCs were excluded from functional connectivity analysis according to this criterion. Then, all data were spatially normalized to MNI space and were smoothed with 4-mm FWHM Gaussian kernel. The images were subsequently detrended to remove linear trends and temporally band-pass filtered (0.01–0.08 Hz) to remove physiological and high frequency noise. Friston 24 motion parameters, global signal, WM signal and CSF signal were regressed out as nuisance signals from the filtered images of each participant. Additionally, to remove the potential confounding effects of micro-movements on resting-state functional connectivity^74^, we applied scrubbing for each motion-outlier. Frames that had >0.5 mm displacement from the previous frame were flagged and removed from subsequent analysis. The mean framewise displacement (FD) in the entire fMRI scans were 0.168 ± 0.05 before scrubbing and 0.156 ± 0.04 after scrubbing. There were no significant differences in mean FD among three groups both before and after scrubbing (*χ* ^2^ = 0.680, p = 0.712, *χ* ^*2*^ = 0.843, p = 0.656). After scrubbing, 97.9 ± 2.6% frames on average remained (at least 155 frames, ~7 min or more), and there was no group difference in the percentage of remaining frames (IGD: 97.5 ± 3.0%; AUD: 97.9 ± 2.7%; HCs: 98.1 ± 2.4%; *χ* ^2^ = 0.126, p = 0.939).

Region showing significantly altered gray matter volume in IGD, the bilateral hippocampus/amygdala, was defined as seed ROI for the resting-state functional connectivity analysis A seed reference time course was obtained by averaging the time series of all voxels in the ROI, then Pearson’s correlation analysis was performed between the seed reference time course and the time series from the whole brain in a voxel-wise way. The correlation coefficients were converted to z values using Fisher’s r-to-z transformation.

### Neuropsychological assessments

To investigate correlations with cortical changes, we conducted comprehensive neuropsychological testing includes following; the verbal fluency test, which tests the ability to generate words in response to a letter cue and assesses cognitive fluency^75^; the Stroop Color and Word Test, which assess sustained and selective attention, cognitive inhibition and working memory^76^; the Trail Making Test, which assesses motor planning and cognitive shifting^77^. Also, three executive function subtests of the Cambridge Neuropsychological Test Automated Battery (CANTAB) were performed; the Intra-Extra Dimensional Set Shift test, a computerised analogue of the Wisconsin Card Sorting Test used to assess the ability to shift and flexibly allocate attention; the Stockings of Cambridge test, which assess spatial planning based upon the Tower of Hanoi test; and the SSP, a visuo-spatial analog of the Digit Span test used to assess working memory capacity^78^.

### Statistical analyses

To evaluate differences between groups in gray matter volume and thickness, statistical tests were performed using a one-way analysis of covariance (ANCOVA) with diagnostic group (IGD, AUD, and HCs) as the factor of interest, followed by post-hoc, two-sample t-test. Due to significant between-group differences in age and IQ, these variables were included as covariates of no interest in both the cortical volume and thickness analyses. Total intracranial volume also included as a covariate of no interest for the VBM analysis. Analyses were corrected for multiple comparisons by means of cluster-size inferences using Monte Carlo simulations. The AlphaSim program in the rest software (http://restfmri.net) was used for the VBM analysis and a precomputed Monte Carlo simulation implemented by FreeSurfer was used for the thickness analysis. The initial cluster-forming threshold employed in the present study was *p* = 0.005, and α = 0.05.

For the functional connectivity analysis, one-sample t-tests were conducted on the individual connectivity maps of the three groups separately, thresholded leniently at a voxel-level threshold of p < 0.05 and a cluster size threshold of more than 491 voxels. To compare the functional connectivity maps between the three groups, statistical tests were performed using an ANCOVA with diagnostic group as the factor of interests and age and IQ included as covariates of no interests followed by post-hoc, two-sample t-test. The significant level was set at p < 0.005 using AlphaSim correction (α = 0.05). The two-sample t-test was masked with a map defined by a one-sample t-test of each relevant group.

Demographic characteristics and scores on neuropsychological tests were evaluated for normality using Shapiro-Wilk. If normality was justified, one-way analyses of variance (ANOVA) was carried out with Tukey’s HSD post-hoc tests. When normality was violated, Kruskal-Wallis tests with Mann–Whitney U post-hoc tests were used to evaluate group differences. Statistical analyses were performed using SPSS 13.0.

Additionally, we examined the relationship between brain cortical data and clinical characteristics in each addiction group (duration of illness; AUDIT-K score, and number of standard drinks for the AUD group; IAT score and mean hours using Internet games per day for the IGD group) and scores on neuropsychological tests showing significant differences between groups using regression analyses of the whole-brain volume or cortical thickness imaging. For regression analyses, an exploratory uncorrected statistical threshold was set at *p* < 0.005, with a minimal cluster size of 100 voxels. In the current study, we focused on the brain regions showing significantly different cortical changes between groups.
